# Soil bacterial and fungal diversity differently correlated with soil biochemistry in alpine grassland ecosystems in response to environmental changes

**DOI:** 10.1038/srep43077

**Published:** 2017-03-06

**Authors:** Yong Zhang, Shikui Dong, Qingzhu Gao, Shiliang Liu, Hasbagan Ganjurjav, Xuexia Wang, Xukun Su, Xiaoyu Wu

**Affiliations:** 1 State Key Laboratory of Water Environment Simulation, School of Environment, Beijing Normal University, Beijing 100875, China; 2 National Plateau Wetland Research Center, Southwest Forestry University, Kunming, 650224, China; 3 Institute of Environment and Sustainable Development in Agriculture, Chinese Academy of Agricultural Sciences, Beijing 100081, China

## Abstract

To understand effects of soil microbes on soil biochemistry in alpine grassland ecosystems under environmental changes, we explored relationships between soil microbial diversity and soil total nitrogen, organic carbon, available nitrogen and phosphorus, soil microbial biomass and soil enzyme activities in alpine meadow, alpine steppe and cultivated grassland on the Qinghai-Tibetan plateau under three-year warming, enhanced precipitation and yak overgrazing. Soil total nitrogen, organic carbon and NH_4_-N were little affected by overgrazing, warming or enhanced precipitation in three types of alpine grasslands. Soil microbial biomass carbon and phosphorus along with the sucrase and phosphatase activities were generally stable under different treatments. Soil NO_3_-N, available phosphorus, urease activity and microbial biomass nitrogen were increased by overgrazing in the cultivated grassland. Soil bacterial diversity was positively correlated with, while soil fungal diversity negatively with soil microbial biomass and enzyme activities. Soil bacterial diversity was negatively correlated with, while soil fungal diversity positively with soil available nutrients. Our findings indicated soil bacteria and fungi played different roles in affecting soil nutrients and microbiological activities that might provide an important implication to understand why soil biochemistry was generally stable under environmental changes in alpine grassland ecosystems.

The relationship between biodiversity and ecosystem functioning is a central issue in ecological research^1^,^2^,^3^. Belowground biodiversity strongly contributes to the maintenance of soil ecosystem functioning and shaping aboveground biodiversity^4^. Bacteria and fungi are the two most abundant groups of soil microbes and are the primary consumers in the soil food web. They play different roles in regulating soil microbiological activities, e.g. specific enzyme activities and soil microbial biomass^5^,^6^,^7^, to mineralize complex organic substances^4^,^8^,^9^ and control the cycling of nutrients and carbon storage in soils^10^. However, the diversity of soil bacteria, fungi and soil microbiological activities and thus soil ecosystem functioning are strongly affected by human activities^5^ and climate change^11^,^12^,^13^.

Alpine regions are sensitive to environmental changes such as climate change^14^,^15^,^16^, but biotic feedbacks, especially from soil microbes that regulate carbon and nitrogen storage in alpine soil ecosystems, are still poorly understood^17^,^18^. The Qinghai-Tibetan plateau (QTP), widely known as the “third pole” of the world, is a typical alpine region dominated by alpine meadow and alpine steppe, but is being impacted by climate change and inappropriate grazing management^19^,^20^. Both short-term and long-term experiments have revealed that the storage of soil carbon and nitrogen and soil microbiological activities are relatively stable in the alpine ecosystems of the QTP under different climate warming and grazing scenarios^21^,^22^. The diversity of microbial functional genes was found to be an important mechanism for maintaining the stability of soil carbon storage in alpine meadow soils under warming conditions^23^. However, a general understanding of the roles of soil microbial diversity in maintaining soil carbon, nitrogen and soil microbiological activities under climate warming in alpine meadows is incomplete. Precipitation is predicted to increase in most regions of the QTP^24^, and extreme climate events could become more frequent. Thus the responses of ecosystems to variable and extreme climate changes should be considered^25^, and a better understanding of these climate change effects on soil microbial diversity and soil functioning of alpine ecosystems is needed.

We conducted this study to explore the relationships between soil microbial diversity and soil biochemistry under conditions of yak overgrazing, climate warming (both stable warming and variable warming) and enhanced precipitation in alpine meadows, alpine steppe and cultivated grasslands of the QTP. The hypotheses of this study were: (1) soil biochemical conditions would be altered by climate changes and yak overgrazing over a short time period (i.e. three years); and (2) soil bacteria and fungi diversity would play different roles in regulating soil biochemistry in response to environmental changes in alpine grassland ecosystems.

## Results

### Soil microbial diversity

**Figure 1:**
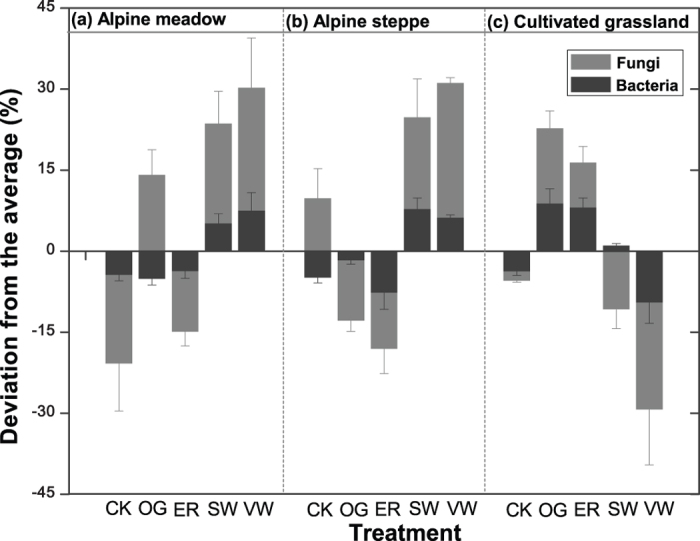
The changes of soil microbial diversity under the fencing without grazing (CK), yak overgrazing (OG), enhanced raining (ER), stable warming (SW) and variable warming (VW) treatments in (**a**) alpine meadow, (**b**) alpine steppe and (**c**) cultivated grassland of the QTP.

In the alpine meadow (AM), the diversity of soil bacteria and fungi were low under the fenced, no-grazing (CK) and enhanced rainfall (ER) treatments, and high under stable warming (SW) and variable warming (VW) treatments (in comparison to the average). The fungi diversity was high, and the bacteria diversity was low under overgrazed (OG) treatment (Fig. 1a). In the alpine steppe (AS), the diversity of bacteria and fungi were low under OG and ER treatments, and high under the SW and VW treatments (Fig. 1b). In the cultivated grassland (CG), the diversity of bacteria and fungi were high under OG and ER treatments, and lower under VW treatment. The diversity of bacteria was high, and the diversity of fungi was low under SW treatment (Fig. 1c). The changes of soil microbial diversity in CK varied among different grassland types (Fig. 1).

**Figure 2:**
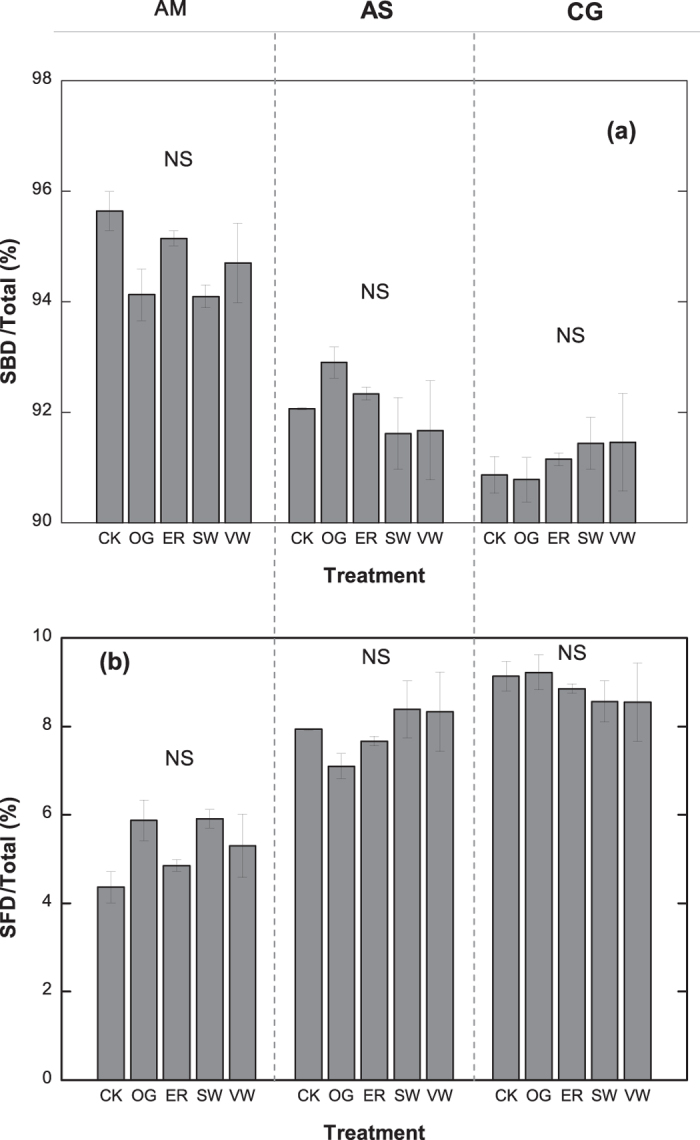
The percentages of (**a**) soil bacterial diversity (SBD) and the total diversity of soil microbes, which was the summation of the diversity of soil bacteria and fungi; (**b**) soil fungal diversity (SFD) and the total diversity of soil microbes in the alpine meadow (AM), alpine steppe (AS) and cultivated grassland (CG) under the fencing without grazing (CK), yak overgrazing (OG), enhanced raining (ER), stable warming (SW) and variable warming (VW) treatments. NS indicates no significant difference. The mean ± s.e. is shown.

The percentage of soil bacterial diversity (SBD) and the soil fungi diversity (SFD) of total microbial diversity was not impacted by yak overgrazing or climate change in all types of grasslands (Fig. 2a,b).

### Soil nutrients

All soil nutrients varied among different grassland types. The soil NO_3_-N changed significantly across different treatments (Table 1 and Supplementary Tables S1 and S2). There were significant interactions between treatment and grassland type on soil nutrients, excluding AP (Table 1). There were no differences in the soil TN, SOC and NH_4_-N among all of the treatments for each type of grasslands (Table 1). Soil NO_3_-N was considerably higher under the ER, SW and VW treatments than the OG treatment in the AM; higher under the SW and VW treatments than the CK treatment in the AS; and higher under the OG treatment than other treatments in the CG (Table 1). The soil AP was not significantly different among treatments in the AM and AS, whereas it was considerably higher under the OG and SW treatments in the CG (Table 1).

### Soil microbiological activities

Two-way ANOVA revealed that the soil microbiological activities, excluding the MBP, varied among different grassland types. The urease activity and MBN were significantly affected by different treatments (Table 2 and Supplementary Tables S1 and S2). There were significant interactions between treatment and grassland type on urease activity, phosphatase activity, MBC and MBN (Table 2). The urease activity was considerably increased under the OG treatment in the AM and the CG. There was no difference in sucrase activity among all of the treatments for each grassland type (Table 2). The phosphatase activity was lower under the VW treatment than the CK in the AM, whereas it was higher under the warming treatments (both SW and VW) than the CK in the AS (Table 2). The MBC was greater under the OG treatment in the AM, whereas it decreased under the ER, SW and VW treatments in the AS. In contrast, an opposite pattern was detected in the CG (Table 2). The highest MBN was detected under the ER treatment, and the lowest under the CK treatment in the AM. In the CG, the highest MBN was detected under the OG treatment and the lowest was detected under the SW treatment. There was no difference in the MBP among all of the treatments in all types of grasslands (Table 2).

### Relationship between soil microbial diversity and soil nutrients

In the AM, obviously positive relationships were observed between the SBD and the TN and SOC under the OG treatment (Table 3). The significant correlations between SBD and NH_4_-N, NO_3_-N and AP were detected under the CK and VW, OG and OG treatments, separately (Table 3). Significantly positive relationships between the SFD and the TN and SOC were detected under the CK and ER treatments (Table 3). The significant correlations between SFD and NH_4_-N, NO_3_-N and AP were detected under the treatments of CK and SW, ER and ER and VW, separately (Table 3). In the AS, there were significantly negative relationships between the SFD and SOC under the treatment of CK. Significantly positive correlations between SFD and NO_3_-N were detected under the CK treatment (Table 3). In the CG, there were significantly positive relationships between the SBD and the TN and SOC under the VW treatment. The SBD and NO_3_-N and AP were significantly correlated under the OG treatment (Table 3). The SFD and TN and SOC were significantly correlated under the treatments of CK and VW. There were significant correlations between SFD and NH_4_-N, NO_3_-N and AP under the CK and ER treatments and the CK and OG treatments, separately (Table 3).

The SBD and SFD showed some opposite relationship modes with soil nutrients in natural grasslands, e.g. the SBD and SFD showed opposite relationship modes with soil NO_3_-N and AP under the OG and ER treatments in the AM (Table 3); the SBD and SFD had opposite relationships with NO_3_-N under nearly all of the treatments in the AS (Table 3).

### Relationships between soil microbial diversity and soil microbiological activities

In the AM, there were significantly positive correlations between SBD and the sucrase and phosphatase activities, whereas there were weakly negative correlations between the SFD and the sucrase and phosphatase activities under the OG treatment (Table 4). Significantly correlations between SFD and urease, sucrase and phosphatase activities were detected under the CK and VW treatments and ER treatment, separately (Table 4). The correlations between soil microbial diversity (both SBD and SFD) and soil microbial biomass were weak, excluding the correlation between SFD and MBN under the VW treatment (Table 4). In the AS, more significant correlations between SFD and soil microbiological activities were detected than the SBD (Table 4). In the CG, there were significantly positive correlations between the SBD and the urease, sucrase and phosphatase activities under the OG and SW treatments, OG treatment and OG and ER treatments, separately (Table 4). The correlations between SFD and soil enzymes activities were nearly all positive and most of them were significant under the treatments of CK, OG, ER, SW and VW (Table 4). The SBD, significantly, negatively correlated with MBC and MBN under the CK and VW treatments and the OG treatment, separately. The SBD was positively correlated with the MBP under the CK treatment (Table 4). The SFD, significantly, correlated with MBC and MBN under the CK and OG treatments and the CK and ER treatments, separately (Table 4).

The SBD and SFD showed some opposite relationship modes with soil microbiological activities under different experimental treatments, e.g. significantly positive relationships were detected between SBD and sucrase and phosphatase activities whereas weakly negative correlations were observed between SFD and sucrase and phosphatase activities under the OG treatment in the AM (Table 4). In addition, the correlations between SBD (or SFD) and MBC was generally opposite to the correlations between SBD (or SFD) and MBN under all the treatments in the AS and the CG (Table 4).

### Regulation process of soil microbial diversity on soil biochemistry

**Figure 3:**
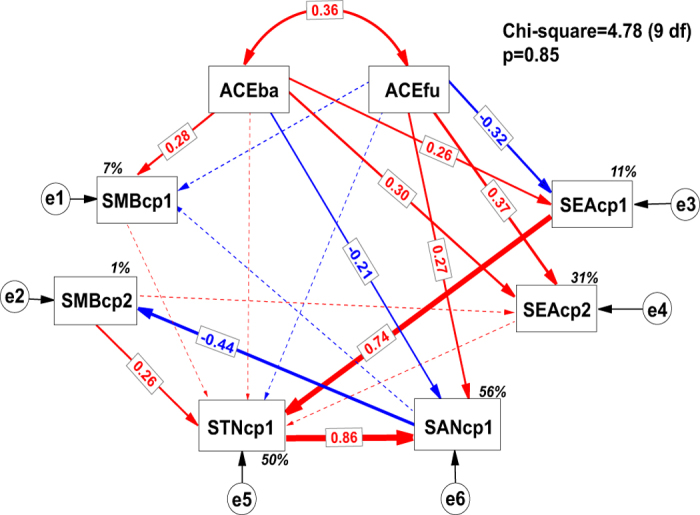
The regulation process of soil microbial diversity on soil biochemistry (n = 135). Soil microbial diversity, i.e. bacterial and fungal diversity (denoted by ACEba and ACEfu) is exogenous variable. The first (SMBcp1) and the second (SMBcp2) components of soil microbial biomass, the first (SEAcp1) and the second (SEAcp2) components of soil enzyme activity, the first component of soil total nutrients (STNcp1) and the first component of soil available nutrients (SANcp1) are endogenous variables. Single-headed arrows indicate causal relationships and double-headed arrows indicate correlation between variables. Numbers on the paths indicate significant standardized path coefficients. The red line indicates positive, and blue line indicates negative effects. The dash lines indicate non-significant effects, which help to improve the quality of the model. The italic percentage on the corner of endogenous variables indicate the variance explained by the model (*R*^2^). The final model fit the data well: comparative fit index (CFI) = 1.0, root mean square error of approximation (RMSEA) < 0.001 and the stability index is 0.23.

The SEM analysis showed a good fit to the data (Fig. 3), indicated by the non-significant χ^2^ value (P = 0.85), high CFI (=1.0), low RMSEA (<0.001) and low stability index (=0.23). The final model explained 7% of the variation in the first component of soil microbial biomass and 1% for the second component. It explained 11% of the variation in the first component of soil enzyme activity and 31% for the second component. The model explained 50% of the variation in the first component of total soil nutrients and 56% of the variation in the first component of soil available nutrients (Fig. 3). Soil bacterial diversity and soil fungal diversity showed opposite relationships with soil microbiological activities, i.e. soil bacterial diversity positively affected soil microbial biomass and soil enzyme activity, while soil fungal diversity negatively affected soil microbial biomass and soil enzyme activity (Fig. 3). Moreover, soil bacteria and fungi showed oppositely relationships with soil nutrients, i.e., soil bacteria negatively affected while soil fungi positively affected the content of soil available nutrients (Fig. 3).

## Discussion

We found that the treatments of OG, SW, VW and ER did not change the soil concentrations of SOC, TN and NH_4_-N or most soil microbial activities in the alpine meadow. This was in agreement with previous results obtained by Wang *et al*.^22^ and Yue *et al*.^23^. We also found that the soil biochemical indicators we measured were generally stable among treatments in the alpine steppe and the cultivated grassland, meaning that not only soil carbon and nitrogen were stable in the alpine grasslands of the QTP under changing environments^26^ but also soil biochemistry. Our findings, however, differed from findings obtained from forest soil ecosystems^27^ implying that different ecosystems may respond differently to climate warming. In addition, we found that concentrations of NO_3_-N and AP, the activity of urease and the MBN increased greatly under the OG treatment in the cultivated grassland, supporting previous work that showed that livestock grazing enhances soil microbial activity and soil nutrient availability in fertilized ecosystems^18^.

In the present study, we found that the ratio of bacteria and fungi diversity to total microbial diversity did not change considerably in the alpine meadow, the alpine steppe or the cultivated grassland under the VW, SW and ER treatments. This property might help to maintain the stability of soil microbial activity^28^ and to sustain the SOC and TN in alpine grassland soils. Based on these observations, we reject our first hypothesis that soil biochemistry (including soil nutrients and microbiological activities) in alpine grasslands would be altered by climate change and yak overgrazing over a short time period.

Previous studies have suggested that soil bacteria play a more important role in mineralizing carbon and nitrogen in fertile habitats whereas soil fungi are more important in infertile systems^29^,^30^. Our results provide supporting evidence for this pattern: firstly, there were more positive relationships between the SFD and soil enzyme activity in relation to SBD among treatments in the alpine steppe as compared to the more nutrient rich alpine meadow^31^; secondly, there were more positive relationships between SBD and soil enzyme activity among treatments in the fertilized cultivated grassland as compared to the alpine meadow (with same soil type as the cultivated grassland).

The roles of soil bacteria and soil fungi in decomposer systems were strongly affected by the quantity and quality of plant litter inputs^10^. Soil fungi are considered more important for breaking down hard-to-decompose matter, such as cellulose and lignin, because of their mycelia networks and better mobility than soil bacteria^10^. In previous studies, we found that livestock grazing decreases but warming increases the proportion of herbivore-preferred plants in alpine grasslands in the QTP^32^,^33^. Herbivore-preferred plant species produce more slowly-decomposable litter^29^,^30^. This implies that soil fungi might be more important decomposers under warming conditions whereas soil bacteria play a more important role when grazing pressure is high. Further evidence that supports this pattern are the mostly positive relationships we observed between the SFD and enzyme activities in the natural alpine grasslands under the warming (VW and SW) and ER treatments whereas the relationships between SBD and enzyme activities were always weak or even negative. Furthermore, more positive relationships between SBD and enzyme activities were detected compared to SFD under the OG treatment. No such patterns were detected in the cultivated grassland which might be due to fertilization effects.

In sum, the relationship between soil bacteria diversity and soil biochemistry was generally opposite to that between soil fungi diversity and soil biochemistry in alpine ecosystems under different environmental conditions. For example, the relationships between SBD and SOC, TN and enzyme activities tended to be positive in the grazing treatment and negative under warming and enhanced precipitation conditions whereas SFD showed an opposite trend. Moreover, the SEM analysis confirmed that soil bacteria and fungi oppositely affected soil microbiological activity and soil nutrients in both direct and indirect ways. In addition, the soil available nutrients negatively affected the soil microbial biomass. The opposite roles of soil bacteria and fungi diversity on soil biochemistry under different environmental conditions along with the negative effects of available nutrients on soil microbial biomass may help to stabilize soil biochemical processes leading to relatively constant nutrient levels and microbial activities in alpine grassland soils over time. These findings support our second hypothesis that soil bacteria and fungi diversity would play different roles in regulating soil biochemical conditions in response to environmental changes in alpine grassland ecosystems.

## Methods and Materials

### Study site description

The study sites were located in Nagqu county (31°26.580′N, 92°1.104′E, at 4 500 m.a.s.l.) and Bange county (31°23.348′N, 92°1.706′E, 4748 m.a.s.l.) in the Tibetan Autonomous Region of China. Alpine meadow (AM), dominated by *Kobresia humilis*, is the typical vegetation in Nagqu County, where the mean annual precipitation is approximately 430 mm and the mean annual temperature is around −1.2 °C (1982–2013). Alpine steppe (AS), dominated by *Stipa purpurea*, is the typical vegetation in Bange County, where the mean annual precipitation is approximately 330 mm and the mean annual temperature is around −0.4 °C (1982–2013). Cultivated grassland (CG) next to an alpine meadow in Nagqu County was planted with *Elymus nutans* in 2009 for a restoration demonstration. Farmyard manure, mixed sheep manure and yak manure, was used to fertilize the cultivated grassland.

### Experimental design and sampling

In 2010, we fenced twelve 4 m × 4 m plots in each of the three grassland types, alpine meadow (AM), alpine steppe (AS) and cultivated grassland (CG) to exclude the animal grazing. These plots were similar in topography and land use histories. There was two-meter distance between each two OTCs to exclude disturbances from other factors^34^, e.g. the cross movement of water, roots and soil temperature among OTCs. Three plots were randomly selected for each of the four treatments: control (CK), stable warming (SW), variable warming (VW) and enhanced raining (ER). The temperature was controlled by facilities commonly used for examining the effects of climatic warming on ecosystems^34^, conical fiberglass open-top chambers (OTCs) with a 1.5 m diameter base, 0.75 m diameter top and 0.4-m height. The temperature in the OTCs under ER was maintained at the ambient temperature outside of the OTCs, and the temperature in the SW treatment OTCs was maintained at a constant temperature of 1.55 °C higher than the ambient temperature using automated mini fans that operated according to temperature probes placed inside and outside the OTCs at 15 cm above the ground. There were no fans in the OTCs of the VW treatments, and the daily air temperature at 5 cm above the soil surface was unevenly elevated to an average temperature of approximately 2.0 °C (range 0~7 °C) over ambient temperatures. In the ER treatment, precipitation was increased by 20% by collecting rainwater with pails and adding it to the OCTs after each rainfall event. For the overgrazing treatment (OG), three plots in the sizes of 50 m × 50 m were selected randomly from each type of grassland outside the fenced ones and were subjected to continual grazing by yaks at an annual stocking rate of 3 animals ha^−1^, which is far higher than the 1–1.5 yak ha^−1^ carrying capacity of alpine grasslands.

We collected 15 soil cores from each grassland type (5 treatments: CK, OG, SW, VW and ER; and 3 replicates (sub-plots) for each treatment) and each soil core was separated into 3 depths: 0~5 cm, 5~10 cm and 10~15 cm. A total of 45 soil cores, a total of 135 soil samples, were collected in July and August of 2013. The soil samples were sealed in polyethylene bags, stored in an icebox and then transported to labs for extraction and other analysis.

### Microbial diversity detection

The microbial DNA was extracted from each of the 135 samples using the E.Z.N.A Soil DNA Kit (Omega Bio-tek, Norcross, GA, U.S.) following the manufacturer’s protocols. For bacteria, the V4-V5 region of the 16 S rRNA gene was amplified using the forward primer 515 F (5′-GTGCCAGCMGCCGCGG-3′) and the reverse primer 907 R (5′-CCGTCAATTCMTTTRAGTTT-3′). For fungi, the ITS rRNA gene was amplified using the primer ITS1 (5′-CTTGGTCATTTAGAGGAAGTAA-3′) and ITS2 (5′-GCTGCGTTCTTCATCGATGC-3′). After purification using the AxyPrep DNA Gel Extraction Kit (Axygen Biosciences, Union City, CA, USA) and quantification using QuantiFluor™ -ST (Promega, Wisconsin, USA), a mixture of amplicons was used for sequencing on the Ilumina MiSeq platform. The processes of quality control and trimming of sequencing reads were performed as described previously^35^.Finally, there were 62,814 paired reads per sample for bacteria, and 36,788 paired reads per sample for fungi.

The operational taxonomic units (OTUs) of bacteria and fungi were defined as sharing >97% sequence identity using the furthest neighbor method (http://www.mothur.org/wiki/Cluster). Then, the species alpha diversity of the microbial community was estimated by the abundance-based coverage estimator (ACE) with OTUs data^36^ using Mother (version v.1.30.1). The total microbial diversity was the summation of the diversity of soil bacteria and fungi. In order to reflect the potentially different roles of soil bacteria and soil fungi in regulating soil biochemistry, the ratio of soil bacteria (or fungi) diversity among the total microbial diversity was presented when to describe diversity changes among different treatments.

### Soil biochemistry measurements

The total nitrogen (TN), soil organic carbon (SOC), NH_4_-N, NO_3_-N and available phosphorus (AP) were determined using methods suggested by Soon and Hendershot^37^. The soil microbial biomass carbon (MBC), nitrogen (MBN) and phosphorus (MBP) were tested using the chloroform fumigation-extraction method^22^. The activities of urease, sucrase and neutral phosphatase, which would benefit the mineralization of soil N and P^22^, were determined by the method of indophenol colorimetry according to Guan^38^ and Tabatabai^39^.

### Statistical analysis

To detect the changes of soil microbial diversity under different treatments, the deviation from the average (DFA) was calculated by:


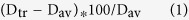


where D_tr_ was the microbial diversity in specific treatment, i.e. CK, OG, ER, SW or VW; D_av_ was the average microbial diversity of all experimental treatments in specific grassland type, i.e. alpine meadow, alpine steppe or cultivated grassland. It means microbial diversity was higher than average if the DFA over zero, vice versa. The DFA of bacteria and fungi was calculated separately.

In each grassland type, one-way ANOVA was employed to test the differences among soil microbial diversity, soil nutrients (TN, SOC, NH_4_-N, NO_3_-N and AP) and soil microbiological activities (enzyme activities and soil microbial biomass) among the different treatments. Two-way ANOVA was conducted to detect the interaction between treatments and grassland types. *Post hoc* tests (Tukey’s) were applied to test the differences among treatments and among grassland types. For each parameter, the homogeneity of variance was tested by using Levene’s test, and necessary transformations were conducted when data did not meet statistical assumptions. These analyses were conducted using IBM SPSS Statistics 19.0.

In order to explore the roles of soil bacteria and fungi diversity on affecting soil biochemistry, a Pearson’s correlation index was calculated between the soil microbial diversity, soil nutrients and soil microbiological activities using IBM SPSS Statistics 19.0. Moreover, the structural equation modeling (SEM), which could detect both direct and indirect effects among variables, was used to obtain a mechanistic understanding of how soil microbial diversity changed soil biochemistry in alpine grassland ecosystems of the QTP. To simplify the model, we reduced the number of soil chemistry variables through Principal Component Analysis (PCA)^40^. And we used the first component if the variance was explained over 50%, else we used more components (until the variance was explained over 50%) to represent soil biochemistry variables (see PCA results in Supplementary Table S3). All paths were considered in the initial model and modified gradually (see Supplementary Fig. S1). Qualified model was indicated by a non-significant *χ*^2^ test (P > 0.05), high comparative fit index (CFI) (>0.95) and low root mean square error of approximation (RMSEA) (<0.05)^40^,^41^. The proposed model was nonrecursive in this study, thus the absolute value of the stability index of the model should be less than 1^42^,^43^. The total 135 samples, i.e. all data collected from three grassland types, were used in SEM procedure. The SEM analyses were performed using AMOS 21.0 (SPSS, Chicago).

## Additional Information

**How to cite this article:** Zhang, Y. *et al*. Soil bacterial and fungal diversity differently correlated with soil biochemistry in alpine grassland ecosystems in response to environmental changes. *Sci. Rep.* **7**, 43077; doi: 10.1038/srep43077 (2017).

**Publisher's note:** Springer Nature remains neutral with regard to jurisdictional claims in published maps and institutional affiliations.

## Supplementary Material

Supplementary Materials

## Figures and Tables

**Table 1:** Soil total nitrogen (TN), soil organic carbon (SOC), soil available nitrogen (NH_4_-N and NO_3_-N) and soil available phosphorus (AP) under the fenced, no-grazing (CK), yak overgrazing (OG), enhanced raining (ER), stable warming (SW) and variable warming (VW) treatments.

Treatment	TN (g/kg)	SOC (g/kg)	NH_4_-N (mg/kg)	NO_3_-N (mg/kg)	AP (mg/kg)
Alpine meadow					
CK	3.98 ± 0.51a	38.13 ± 5.31a	5.94 ± 0.84a	54.26 ± 4.71ab	9.75 ± 0.96a
OG	3.22 ± 0.27a	31.68 ± 3.09a	4.12 ± 0.54a	36.91 ± 7.59b	8.51 ± 0.89a
ER	4.04 ± 0.70a	42.46 ± 7.69a	5.95 ± 0.56a	77.30 ± 5.61a	9.30 ± 1.32a
SW	4.52 ± 0.58a	48.28 ± 6.27a	7.12 ± 2.00a	88.88 ± 11.81a	10.97 ± 1.39a
VW	4.14 ± 0.74a	42.16 ± 7.09a	12.13 ± 3.47a	78.70 ± 10.29a	10.27 ± 1.29a
Alpine steppe					
CK	2.83 ± 0.16a	23.38 ± 0.67a	23.24 ± 5.07a	22.63 ± 0.45b	10.75 ± 0.77a
OG	3.87 ± 0.31a	36.05 ± 1.77a	26.87 ± 7.17a	32.19 ± 4.69ab	14.48 ± 1.15a
ER	3.67 ± 0.30a	31.82 ± 2.66a	28.71 ± 4.51a	35.73 ± 3.55ab	12.31 ± 1.43a
SW	3.04 ± 0.17a	25.82 ± 1.04a	24.41 ± 4.18a	42.29 ± 1.54a	10.63 ± 0.56a
VW	3.53 ± 0.14a	29.37 ± 1.01a	19.99 ± 1.79a	44.87 ± 3.87a	12.76 ± 0.95a
Cultivated grassland					
CK	1.88 ± 0.12a	22.03 ± 1.18a	6.22 ± 0.57a	26.66 ± 2.67b	5.79 ± 0.69b
OG	3.32 ± 0.38a	37.49 ± 4.21a	9.19 ± 1.26a	59.44 ± 4.17a	12.08 ± 2.09a
ER	2.37 ± 0.11a	26.73 ± 0.91a	7.02 ± 0.93a	27.96 ± 2.89b	9.45 ± 0.99ab
SW	2.59 ± 0.23a	27.08 ± 2.10a	13.49 ± 3.95a	27.37 ± 0.90b	11.04 ± 2.11a
VW	1.97 ± 0.19a	22.24 ± 2.59a	8.83 ± 0.55a	23.45 ± 2.20b	9.26 ± 0.92ab
Treatment	1.13	0.83	1.47^†^	5.91^†,^***	2.44
Grassland type	24.22***	23.55***	63.90^†,^***	70.51^†,^***	7.77**
Treatment* Grassland type	2.33*	2.69*	2.30^†,^*	13.43^†,^***	1.98

In each grassland type, different letters represent significant differences at *p* < 0.05 tested by one-way factorial ANOVA (n = 3). The mean ± s.e. is shown. Two-way ANOVA was conducted to detect the interaction between treatments and grassland types. *F* value is listed for the two-way ANOVA. *P < 0.05; **P < 0.01; ***P < 0.001. ^†^Log-transformation.

**Table 2:** Soil microbiological activities under the treatments with fencing without grazing (CK), yak overgrazing (OG), enhanced raining (ER), stable warming (SW) and variable warming (VW).

Treatment	Urease activity (mg NH_4_^+^/g·24 h)	Sucrase activity (mg glucose/g·24 h)	Phosphatase activity (mg phenol/g·24 h)	MBC (mg/kg)	MBN (mg/kg)	MBP (mg/kg)
Alpine meadow						
CK	0.36 ± 0.01b	45.19 ± 4.41a	12.58 ± 0.27a	246.30 ± 45.52b	116.05 ± 22.59b	17.24 ± 1.60a
OG	0.79 ± 0.09a	53.27 ± 3.89a	12.28 ± 0.23ab	507.47 ± 51.82a	188.59 ± 2.16ab	9.82 ± 0.70a
ER	0.28 ± 0.03b	56.59 ± 4.25a	11.29 ± 0.20ab	297.91 ± 39.16b	247.53 ± 26.84a	15.59 ± 4.07a
SW	0.40 ± 0.02b	55.98 ± 4.99a	11.00 ± 0.87ab	229.18 ± 20.19b	196.30 ± 25.05ab	17.98 ± 2.07a
VW	0.41 ± 0.08b	46.47 ± 2.99a	10.13 ± 0.45b	232.46 ± 3.97b	180.25 ± 17.18ab	18.21 ± 2.41a
Alpine steppe						
CK	0.27 ± 0.01a	8.81 ± 0.85a	9.07 ± 0.29b	538.48 ± 29.00a	197.53 ± 26.89a	12.54 ± 1.70a
OG	0.39 ± 0.03a	13.57 ± 1.54a	10.49 ± 0.12ab	417.33 ± 41.07ab	199.38 ± 6.79a	13.51 ± 4.97a
ER	0.40 ± 0.09a	9.18 ± 1.49a	10.22 ± 0.44ab	368.82 ± 35.77b	205.56 ± 30.32a	18.44 ± 5.10a
SW	0.61 ± 0.19a	10.68 ± 1.70a	11.31 ± 0.36a	344.91 ± 20.35b	150.62 ± 21.95a	19.13 ± 3.33a
VW	0.45 ± 0.09a	11.89 ± 1.12a	10.68 ± 0.28a	283.05 ± 6.70b	224.69 ± 8.97a	18.40 ± 3.46a
Cultivated grassland						
CK	0.84 ± 0.05b	36.85 ± 2.93a	8.00 ± 0.14a	243.76 ± 3.08b	239.88 ± 3.74ab	17.85 ± 4.26a
OG	1.38 ± 0.05a	48.69 ± 3.00a	8.08 ± 0.18a	227.89 ± 38.55b	283.22 ± 36.35a	17.55 ± 7.45a
ER	1.02 ± 0.10b	34.69 ± 1.55a	8.83 ± 0.14a	409.75 ± 11.14a	201.57 ± 3.22ab	17.85 ± 1.19a
SW	0.90 ± 0.01b	30.71 ± 3.30a	9.45 ± 0.23a	465.43 ± 17.46a	168.17 ± 9.17b	16.04 ± 7.99a
VW	0.38 ± 0.06c	32.42 ± 8.12a	9.44 ± 0.03a	420.19 ± 34.55a	237.65 ± 24.48ab	12.71 ± 2.78a
Treatment	10.75^‡,^***	2.51^†^	1.58^‡^	2.20	3.68*	0.46
Grassland type	41.79^‡,^***	218.92^†,^***	37.77^‡,^***	10.35***	5.16*	0.04
Treatment* Grassland type	6.20^‡,^***	1.39^†^	2.47^‡,^*	17.54***	3.80**	0.56

In each grassland type, different letters represent significant differences at *p* < 0.05 tested by a one-way factorial ANOVA (n = 3). The mean ± s.e. is shown. Two-way ANOVA was conducted to detect the interaction between treatments and grassland types. *F* value is listed for the two-way ANOVA. *P < 0.05; **P < 0.01; ***P < 0.001. ^†^Log-transformation. ^‡^Arctan square-root transformation.

**Table 3:** The correlation index between soil microbial diversity (soil bacteria diversity, SBD; and soil fungi diversity, SFD) and soil nutrients under no grazing (CK), yak overgrazing (OG), enhanced raining (ER), stable warming (SW) and variable warming (VW) treatments (n = 9).

Treatments	SBD & TN	SBD & SOC	SBD & NH_4_-N	SBD & NO_3_-N	SBD & AP	SFD & TN	SFD & SOC	SFD & NH_4_-N	SFD & NO_3_-N	SFD & AP
Alpine meadow										
CK	0.57	0.49	0.87***	0.29	0.20	0.67**	0.61*	0.77**	0.51	0.29
OG	0.85***	0.86***	0.53	0.68**	0.87***	−0.21	−0.24	−0.14	−0.46	0.04
ER	−0.06	−0.07	−0.40	−0.24	−0.09	0.79**	0.82***	−0.52	0.85***	0.85***
SW	−0.31	−0.24	−0.10	−0.37	−0.10	−0.23	−0.18	0.83***	−0.43	−0.22
VW	0.02	0.06	0.77**	0.03	0.23	0.32	0.47	0.45	0.36	0.58*
Alpine steppe										
CK	−0.06	−0.24	−0.24	0.12	0.03	−0.10	−0.59*	0.24	0.73**	0.03
OG	−0.30	−0.01	0.30	0.05	0.03	−0.39	−0.24	−0.49	−0.12	−0.53
ER	−0.34	−0.35	0.43	−0.25	−0.26	−0.27	−0.33	−0.03	0.38	0.15
SW	−0.26	−0.21	0.04	−0.28	−0.20	0.25	0.39	0.29	0.14	−0.28
VW	0.27	0.18	0.39	−0.29	−0.16	0.10	0.14	−0.44	0.52	0.10
Cultivated grassland										
CK	0.13	0.21	0.20	0.26	−0.05	0.73**	0.81***	0.77**	0.70**	0.76**
OG	0.53	0.45	0.24	0.58*	0.59*	0.23	0.22	0.15	0.77**	0.76**
ER	0.01	0.00	0.32	0.41	0.18	−0.13	−0.20	0.63*	0.41	0.31
SW	0.20	0.28	−0.15	−0.07	−0.15	0.27	0.33	−0.19	−0.01	−0.09
VW	0.67**	0.75**	−0.29	0.28	−0.16	0.86***	0.90***	−0.11	0.46	0.01

*P < 0.1; **P < 0.05; ***P < 0.01.

**Table 4:** The correlation index between soil microbial diversity (soil bacteria diversity, SBD; and soil fungi diversity, SFD) and soil microbiological activities under CK, yak overgrazing (OG), enhanced raining (ER), stable warming (SW) and variable warming (VW) treatments (n = 9).

Treatments	SBD & MBC	SBD & MBN	SBD & MBP	SBD & urease	SBD & sucrase	SBD & phosphatase	SFD & MBC	SFD & MBN	SFD & MBP	SFD & urease	SFD & sucrase	SFD & phosphatase
Alpine meadow												
CK	0.40	0.07	0.48	0.53	0.32	0.25	0.51	−0.15	0.41	0.65*	0.48	0.51
OG	−0.39	0.28	0.49	−0.45	0.70**	0.89***	−0.32	−0.21	−0.36	−0.22	−0.20	−0.08
ER	0.19	0.37	−0.43	−0.11	0.01	−0.34	−0.16	−0.12	−0.15	0.49	0.64*	0.68**
SW	0.29	0.45	−0.27	−0.24	−0.17	−0.09	0.42	0.09	0.31	−0.13	0.05	0.17
VW	0.26	0.51	−0.13	0.17	0.15	−0.23	0.42	0.76**	−0.34	0.59*	0.38	−0.05
Alpine steppe												
CK	0.14	−0.16	0.08	0.03	0.67**	0.46	0.50	−0.57	−0.67*	0.76**	−0.06	0.91***
OG	0.10	−0.38	0.20	0.47	0.26	0.38	0.55	−0.10	0.08	0.48	0.29	−0.06
ER	−0.19	0.56	0.11	0.41	0.56	−0.06	−0.03	0.26	−0.45	0.33	−0.08	0.59*
SW	−0.33	0.56	0.64*	0.20	−0.41	0.00	0.00	0.29	0.36	0.55	0.37	0.17
VW	0.15	−0.16	0.10	0.02	0.31	−0.20	0.06	−0.11	0.24	0.60*	0.68*	0.86***
Cultivated grassland												
CK	−0.59*	0.02	0.63*	0.27	0.28	−0.11	−0.94***	−0.59*	0.26	0.71**	0.68*	0.51
OG	0.35	−0.63*	−0.15	0.69**	0.76**	0.67**	0.60*	−0.49	0.06	0.91***	0.76**	0.78**
ER	0.30	−0.44	0.15	0.10	0.12	0.66*	0.17	−0.62*	0.16	0.01	0.18	0.62*
SW	−0.54	0.12	0.22	0.81***	0.15	0.31	−0.38	0.07	0.06	0.82***	0.45	0.55
VW	−0.80***	0.31	0.26	−0.01	0.55	−0.21	−0.58	0.31	0.01	0.41	0.85***	−0.10

*P < 0.1; **P < 0.05; ***P < 0.01.
