# Supplementary material for: Soil bacterial and fungal diversity differently correlated with soil biochemistry in alpine grassland ecosystems in response to environmental changes
Source: Sci Rep. 2017 Mar 6;7:43077. doi: 10.1038/srep43077 (PMC5338028; doi:10.1038/srep43077)
Supplement: Supplementary Materials [file srep43077-s1.pdf]

Soil bacterial and fungal diversity differently correlated with soil biochemistry in alpine grassland ecosystems in response to environmental changes

Yong Zhang[1], Shikui Dong[1,2*], Qingzhu Gao[3*], Hasbagan Ganjurjav[3], Xuexia Wang[3], Xukun Su[1], Xiaoyu Wu[1]

Supplementary Table S1. The result of *Post hoc* tests (Tukey's) for soil total nitrogen (TN), soil organic carbon (SOC), soil available nitrogen (NH_4-N and NO_3-N), soil available phosphorus (AP), soil microbial biomass carbon (MBC), soil microbial biomass nitrogen (MBN), soil microbial biomass phosphorus (MBP) and soil enzyme activities among the CK, yak overgrazing (OG), enhanced raining (ER), stable warming (SW) and variable warming (VW) treatments. Different letters represent significant differences at $p < 0.05$. The mean ± s.e. is shown.

	TN (g/kg)	SOC (g/kg)	NH_4-N †	NO_3-N †	AP (mg/kg)	MBC (mg/kg)	MBN (mg/kg)	MBP (mg/kg)	Urease‡	Sucrase†	Phosphatase‡
CK	2.90 ± 0.32	32.15 ± 4.10	0.96 ± 0.10	1.50 ± 0.06^{b}	7.35 ± 0.68^{b}	342.84 ± 51.34	184.49 ± 20.83^{ab}	15.88 ± 1.63	0.59 ± 0.04^{c}	1.39 ± 0.11	2.27 ± 0.07
OG	3.47 ± 0.24	37.23 ± 2.53	1.00 ± 0.12	1.61 ± 0.05^{ab}	9.74 ± 0.80^{a}	384.23 ± 46.74	223.73 ± 18.38^{a}	13.63 ± 2.82	0.72 ± 0.05^{a}	1.51 ± 0.10	2.32 ± 0.06
ER	3.28 ± 0.29	37.68 ± 3.99	1.02 ± 0.11	1.63 ± 0.07^{a}	8.63 ± 0.69^{ab}	358.83 ± 22.62	218.22 ± 13.84^{ab}	17.29 ± 1.96	0.61 ± 0.05^{bc}	1.41 ± 0.12	2.31 ± 0.04
SW	3.37 ± 0.36	36.84 ± 4.16	1.09 ± 0.10	1.67 ± 0.08^{a}	9.07 ± 0.67^{ab}	364.51 ± 35.45	171.70 ± 11.99^{b}	17.72 ± 2.61	0.67 ± 0.03^{ab}	1.41 ± 0.11	2.35 ± 0.04
VW	3.21 ± 0.37	35.30 ± 4.33	1.10 ± 0.07	1.63 ± 0.08^{a}	8.97 ± 0.63^{ab}	311.90 ± 29.85	214.20 ± 12.52^{ab}	16.44 ± 1.73	0.56 ± 0.02^{c}	1.40 ± 0.10	2.25 ± 0.07

† Log-transformation. ‡ Artan square-root transformation.

Supplementary Table S2. The result of *Post hoc* tests (Tukey's) for soil total nitrogen (TN), soil organic carbon (SOC), soil available nitrogen (NH_4-N and NO_3-N, mg/kg), soil available phosphorus (AP), soil microbial biomass carbon (MBC), soil microbial biomass nitrogen (MBN), soil microbial biomass phosphorus (MBP) and soil enzyme activities among alpine meadow (AM), alpine steppe (AS) and cultivated grassland (CG). Different letters represent significant differences at $p < 0.05$. The mean ± s.e. is shown.

	TN (g/kg)	SOC (g/kg)	NH_4-N †	NO_3-N †	AP (mg/kg)	MBC (mg/kg)	MBN (mg/kg)	MBP (mg/kg)	Urease‡	Sucrase†	Phosphatase ‡
AM	3.98 ± 0.16^{a}	46.42 ± 2.74^{a}	0.80 ± 0.05^{b}	1.80 ± 0.04^{a}	8.13 ± 0.38^{b}	302.67 ± 31.37^{b}	185.74 ± 13.70^{b}	15.77 ± 1.24	0.58 ± 0.02^{b}	1.71 ± 0.02^{a}	2.43 ± 0.03^{a}
AS	3.34 ± 0.15^{b}	32.06 ± 1.56^{b}	1.37 ± 0.04^{a}	1.54 ± 0.03^{b}	10.15 ± 0.53^{a}	390.52 ± 25.42^{a}	195.56 ± 10.34^{ab}	16.41 ± 1.66	0.56 ± 0.02^{b}	1.02 ± 0.03^{c}	2.33 ± 0.02^{b}
CG	2.42 ± 0.23^{c}	29.03 ± 2.62^{b}	0.93 ± 0.04^{b}	1.49 ± 0.04^{b}	7.97 ± 0.58^{b}	353.40 ± 27.83^{a}	226.10 ± 12.87^{a}	16.40 ± 2.11	0.74 ± 0.03^{a}	1.55 ± 0.03^{b}	2.13 ± 0.04^{c}

† Log-transformation. ‡ Artan square-root transformation.

Supplementary Table S3. Component matrix (only the first and the second components) detected by Principal Component Analysis (PCA) for different soil indexes.

Indexes		Component1	Component2
Soil microbial biomass	Biomass carbon	-0.78	-0.13
	Biomass nitrogen	0.19	0.92
	Biomass phosphorus	0.71	-0.39
Variance explained		38.07%	34.21%
Soil enzyme activity	Urease	0.40	0.84
	Sucrase	0.89	0.09
	Phosphatase	0.64	-0.66
Variance explained		43.63%	40.19%
Soil total nutrients	Nitrogen	0.98	-0.07
	Soil total carbon	0.97	-0.22
	Soil organic carbon	0.96	-0.17
	Phosphorus	0.52	0.85
Variance explained		70.42%	27.24%
Soil available nutrients	NH_4-N	0.35	0.91
	NO_3-N	0.79	-0.51
	Available phosphorus	0.92	0.09
Variance explained		50.93%	38.22%

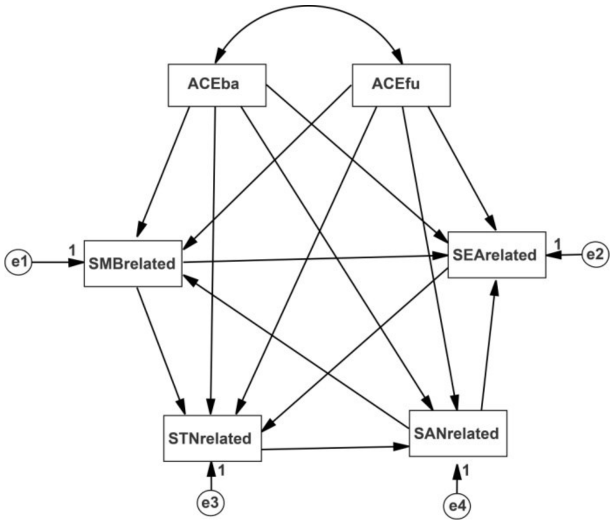

Supplementary Figure S1. The conceptual model of the path analysis. ACEba represents the richness of soil bacteria; ACEfu represents the richness of soil fungi; SMBrelated represents the index that could reflect soil microbial biomass; SEArelated represents the index that could reflect soil enzyme activity; STNrelated represents the index that could reflect soil total nutrients; SANrelated represents the index that could reflect soil available nutrients.
